# Rethinking the serological threshold for onchocerciasis elimination

**DOI:** 10.1371/journal.pntd.0006249

**Published:** 2018-03-15

**Authors:** Katherine M. Gass

**Affiliations:** Neglected Tropical Disease Support Center, The Task Force for Global Health, Decatur, Georgia, United States of America; Imperial College London, Faculty of Medicine, School of Public Health, UNITED KINGDOM

A measurement is only as precise as the ruler. So too in public health is our ability to detect disease or its absence only as accurate as the diagnostic tool. This tension between a public health goal and the quality of available diagnostics is currently playing out on a global scale with onchocerciasis.

Onchocerciasis, also known as river blindness, is a disease primarily of the skin and eyes that is caused by a parasitic worm. The disease can be controlled and prevented through long-term, once-yearly mass drug administration (MDA) with ivermectin and has recently become one of 11 neglected tropical diseases that the World Health Organization (WHO) has slated for elimination [1]. However, elimination of onchocerciasis first requires that country programs can determine when it is safe to stop MDA and transition to a period of post-treatment surveillance. To provide such guidance, WHO recently produced guidelines for “Stopping Mass Drug Administration and Verifying Elimination of Human Onchocerciasis,” in which it is stated that treatment-stopping decisions should be based on entomological evaluation to detect infection in the vector, black flies, and serological evaluation in humans to detect the presence of antibodies to *Ochocerca volvulus* Ov16 antigen [2].

According to these guidelines, the serological threshold is an Ov16 antibody prevalence of less than 0.1% among children under 10 years of age. This low threshold was guided by results of observational studies in Guatemala and Uganda and chosen to be highly conservative [3–5]. With many national onchocerciasis programs in Africa nearing the number of years of recommended MDA and preparing to apply this guidance in a programmatic setting, it is imperative that we assure that reaching this serological threshold is epidemiologically feasible.

The first question to ask is whether we have a diagnostic tool that is sufficiently specific to define such a threshold. No test can detect a threshold that is less than the number of false positives it is likely to produce. Practically speaking, this means the lowest threshold one can reliably measure must exceed one minus the specificity. The two most common diagnostic tools available for detecting Ov16 are enzyme-linked immunosorbent assays (ELISA) and a lateral flow-based assay, available as a point-of-care rapid diagnostic test (RDT). Published data on the specificity of Ov16 ELISA ranges from 97% to >99.9% [6–8]. Laboratory testing of the RDT demonstrated an Ov16 specificity of 97%–98% [9, 10]. Because being successful at detecting when <0.1% prevalence of Ov16 has been reached requires a diagnostic tool that reliably achieves >99.9% specificity, the current tools are clearly not yet up to the task.

The sensitivity of the diagnostic tools is similarly important to consider. If the tool has poor sensitivity, then some positive individuals will go undetected. Clinical studies suggest that 15%–25% of people may have some genetic restriction that prevents them from mounting an immune response to Ov16 antigen [11]. This suggests that any measure of Ov16 serology will systematically miss approximately 20% of infected individuals. In the case of MDA-stopping decisions, a less sensitive tool means that evaluated areas have an added risk of falling below the prevalence-stopping threshold and of stopping MDA prematurely (unless other compensations were made, such as increasing sample size).

Finally, there is the practical issue of sample size. If we had a test with perfect accuracy (100% sensitivity and specificity), the minimum sample size required to detect an antibody prevalence of less than 0.1% Ov16 with 95% confidence that we will correctly identify those areas that are above the stopping threshold (i.e., a Type 1 error rate of α = 5%) is 2,995 children. The critical value associated with this decision rule, i.e., the maximum number of observed positive results that is consistent with the threshold, is zero. In other words, only if all 2,995 children test negative could one conclude that the true prevalence is likely below 0.1%; a single positive test would cause the area to “fail” (exceed the threshold). While this sample size will enable programs to successfully identify areas that should continue MDA, it will often fail to identify areas that may be eligible to stop. The ability to correctly identify areas that should “pass” (fall below the threshold) is referred to as power. Using the binomial distribution, areas where the true Ov16 antibody prevalence is half the threshold (e.g., 0.05%) are likely to find zero positive out of 2,995 children tested only 22% of the time. To put this in programmatic context, 78 out of 100 assessment areas that have successfully driven the prevalence of onchocerciasis below the 0.1% threshold will still fail the assessment and continue ivermectin distribution. Many statisticians would consider an assessment with only 22% power to be unacceptable; indeed, for lymphatic filariasis (LF), another neglected tropical disease with a similar treatment and assessment strategy, 75% power was deemed a reasonable balance of programmatic feasibility and statistical inference.

A simple way to increase power is to increase the sample size. However, to achieve close to 75% power for detecting a threshold of 0.1%, maintaining a 5% chance of Type 1 error, requires sampling 15,700 children. A sample size of this magnitude is likely to be unattainable for country programs. While a finite population adjustment will reduce the sample size when the assessment area is small, the figure remains prohibitively large. Moreover, this is before taking into account the design effect (DEFF), a variance adjustment that is necessary to allow for correlations within clusters of observations. Incorporating a DEFF of 2, as was done for the similar assessment with LF [12], would double the current sample size projections.

In short, the sample size required to adequately measure a threshold of 0.1% Ov16 antibody is neither possible with the current diagnostic tools nor feasible for national onchocerciasis programs. Nonetheless, numerous countries with endemic foci under treatment with ivermectin for more than a dozen years are now in need of assessing whether or not transmission persists [13]. For these countries, it is imperative that a provisional, measurable treatment-stopping threshold be set.

Any revision to the current threshold guidelines should be rooted in the transmission dynamics of the parasite, as it would be inappropriate for programs to stop MDA before the transmission breakpoint has been achieved. However, until more evidence can be generated from post-MDA settings, we need to rely on models to estimate the most likely transmission breakpoints in terms of Ov16 antibody prevalence. Recent results from ONCHOSIM, an established mathematical model for simulating transmission and control of onchocerciasis, suggest that an Ov16 prevalence of ≤2% in children under 10 years of age is associated with a high probability of elimination in most situations [14]. Experience from the LF transmission assessment survey suggests that measuring such a 2% threshold is feasible for country programs to implement with the resources available [15].

While an Ov16 threshold of <2% may ultimately prove to be the most appropriate serological criterion for stopping MDA, it would be premature to revise WHO guidelines at this time. Both the Ov16 ELISA and RDT diagnostic tools are presently undergoing refinements. The results of field validation studies that compare these tools’ performance in low-prevalence settings are needed in order to calibrate a serological threshold to the accuracy of the tests. It should be noted that much of the empirical evidence for serological thresholds comes from the experience of the Onchocerciasis Elimination Program for the Americas (OEPA). Though the OEPA data are informative, the epidemiological characteristics of onchocerciasis transmission are very different in Africa [16]. Additional data from near-elimination settings in Africa are needed to determine the most appropriate serological endpoint. Furthermore, because of the challenges presented by black fly migration, expansive transmission foci, and the potential for residual “hot spots” of transmission where ivermectin coverage is low, new sampling strategies may be required. This will likely involve a combination of purposive and systematic random sampling in order to have greater confidence in stopping-MDA decisions.

Finally, it is important to recognize that longitudinal data and transmission models suggest that both treatment duration and transmission breakpoints depend on baseline endemicity [14, 17]. To address this variable, one option is to recommend different serological thresholds for settings with low, moderate, and high precontrol endemicity. Conversely, because the use of multiple thresholds risks adding confusion—particularly in countries with varying baseline endemicities—and because many settings may not have good baseline data, the lowest serological threshold could be applied universally. Whatever the decision, it is important that any revision to the definition of the serological threshold respect the heterogeneity of transmission intensities that exists across Africa.

In order to eliminate onchocerciasis, it is imperative that country programs be able to measure when it is safe to stop treatment. The current threshold of less than 0.1% Ov16 serology in children under 10 years of age represents a restrictive target that is neither practical for resource-limited countries nor possible with the current diagnostic tools. That some countries, mainly in the Americas, have been able to achieve this target is commendable but not representative of what will be achievable in most African settings. Moving forward, we must revise the current serological threshold for stopping MDA and develop an assessment strategy that reflects the accuracy of the latest diagnostic tools, is feasible for implementation by programs, and respects the parasite’s transmission dynamics. Only with a well-thought-out sampling strategy and revised decision rules will it be possible to increase the likelihood that areas where transmission has been interrupted are recognized and allowed to stop MDA while minimizing the risk that MDA is stopped prematurely in places where transmission is ongoing. Such revisions will not only help set up country programs for success; they will also protect the global goal of onchocerciasis elimination.
